# An Intensive Lifestyle Intervention to Treat Type 2 Diabetes in the Republic of the Marshall Islands: Protocol for a Randomized Controlled Trial

**DOI:** 10.3389/fnut.2019.00079

**Published:** 2019-06-05

**Authors:** Brenda C. Davis, Humaira Jamshed, Courtney M. Peterson, Joan Sabaté, Ralph D. Harris, Rohit Koratkar, Jamie W. Spence, John H. Kelly

**Affiliations:** ^1^Brenda Davis Nutrition Consultation Services, Kelowna, BC, Canada; ^2^Department of Nutrition Sciences, University of Alabama at Birmingham, Birmingham, AL, United States; ^3^School of Public Health, Center for Nutrition, Lifestyle, and Disease Prevention, Loma Linda University, Loma Linda, CA, United States; ^4^The Meridian Senior Retirement Center, San Marcos, CA, United States; ^5^Canvasback Missions, Inc., Benicia, CA, United States; ^6^Department of Preventive Medicine, School of Medicine, Loma Linda University, Loma Linda, CA, United States; ^7^Black Hills Lifestyle Medicine Center, Hermosa, SD, United States

**Keywords:** type 2 diabetes, lifestyle intervention, plant-based diet, exercise, Republic of the Marshall Islands

## Abstract

**Background:** The Republic of the Marshall Islands has the highest prevalence of type 2 diabetes (T2D) in the world, with the country's rapid rise of T2D attributed to its reliance on imported and refined foods laden with salt, sugar, and fat. As much as lifestyle factors can increase the risk of T2D, they can also reverse or treat the disease, with multiple studies demonstrating that plant-based diets and/or moderate exercise improve glycemic control and cardiovascular risk factors in T2D patients.

**Objective:** We therefore tested the hypothesis that a community-based, intensive, plant-rich lifestyle intervention with exercise is more effective for treating and managing T2D in the Republic of the Marshall Islands than the standard of diabetes care.

**Methods:** Building on a successful lifestyle program used at the Guam Seventh-day Adventist Clinic, we conducted a randomized controlled trial to test the effectiveness of an intensive lifestyle intervention involving a plant-rich diet and moderate exercise or the standard of care in T2D patients for 24 weeks. In this manuscript, we describe the clinical trial protocol, including the rationale, design, and methods of the clinical trial and the lifestyle program. The lifestyle intervention included a step-wise, intensive 12-week program of counseling and instruction on healthy eating, exercise, and stress management. The prescribed diet focused on high-fiber, whole plant foods, with foods grouped into a four-tiered system. The lifestyle intervention also involved hands-on cooking classes, meals prepared for participants, and group exercise classes—all tailored to be culturally appropriate. The study's main endpoints were glycemic control and cardiovascular disease risk factors.

**Discussion:** The present study is the first randomized clinical trial conducted in the Republic of the Marshall Islands and the first lifestyle intervention trial conducted in Micronesia. The results of this study will help guide future medical care for indigenous populations in the Pacific Islands and will also shed light on how to effectively design and deliver intensive lifestyle interventions to treat and manage diabetes.

**Clinical Trials Registration:** www.ClinicalTrials.gov; identifier NCT03862963

## Introduction

Diabetes is a rising epidemic. More than 420 million people worldwide have the disease (1), and new estimates suggest that diabetes now afflicts about 9.4% of Americans (2). Yet, in some countries, such as within the Pacific Islands and Micronesia, the prevalence of diabetes among adults is as high as 25–31% (3).

The Republic of the Marshall Islands (RMI) has the highest prevalence of diabetes in the world, where the disease afflicts almost one-third (31.4%) of all adults aged 18–99 years (3). The explosion in diabetes rates in the RMI is a relatively recent phenomenon. In the 1950's, only three people in the RMI were known to have the disease, according to an oral report by an RMI health official in 2005. By the 1990's, the prevalence had reached 30% (4). Now, more than half of all hospital admissions in the capital city of Majuro are due to diabetes (5), and 60% of deaths in adults under the age of 60 years are attributed to the disease and its related comorbidities (5). The epidemic has been attributed to the growing population, which has strained supplies of healthier indigenous foods and increased the reliance on imported goods such as flour, rice, and foods high in salt, sugar, and fats (6).

To combat the growing global diabetes epidemic, the American Diabetes Association has called for increased use of lifestyle interventions as part of primary care (7). Indeed, dietary interventions can lower hemoglobin A1c (HbA1c) by 0.5–2.0% (7), with several trials reporting values between 1.0 and 3.4% (8–20) as well as reductions in hyperglycemic medications and/or even T2D remission (8, 10–12, 15, 17, 19, 21–27). In particular, several trials suggest that vegetarian and vegan low-fat diets are superior to conventional diets for lowering glucose, HbA1c, and insulin levels; decreasing diabetes medication dosages; and reducing diabetes complications, such as neuropathy, in T2D patients (12, 19, 20, 22, 26–40). This is corroborated by prospective cohort studies, such as the Nurses' Health Study and Adventist Health Study-2, which report that plant-based diets reduce the risk of developing type 2 diabetes by about half (41–45). Moreover, among populations such as the Seventh-day Adventists, the prevalence of diabetes among vegans is as low as 0.22 times that among non-vegetarians (45, 46). Plant-based diets have also been found to improve lipids, renovascular health, and oxidative stress levels in adults with T2D more effectively than conventional diets (12, 19, 22, 26, 28, 31–35, 38, 47). Plant-based diets likely mediate these effects through multiple mechanisms (47–49), including through improvements in body weight and visceral fat [e.g., (32, 36, 50, 51)]; insulin sensitivity and beta-cell function (32, 39, 52, 53); incretins and gastrointestinal hormones (39, 52); oxidative stress (32); phytochemical intake [including polyphenols and plant sterols (49, 54)]; fiber and prebiotic intake, which positively modulate gut microbiota (55); lower oxidant intake (such as heme iron) (49); and increased well-being (37). Lifestyle interventions that combine plant-diets with regular exercise may be even more effective. Coupling a plant-based diet with exercise significantly reduces the incidence of diabetes in glucose-impaired individuals (56, 57), and clinical trials show that the combination is effective for treating diabetes, cardiovascular disease, obesity, and other chronic conditions (32, 58–64). To date, however, only one previous clinical trial has combined a plant-rich diet with exercise for treating T2D (65).

Despite the promise of such lifestyle interventions, implementing them in indigenous populations such as the RMI presents unique challenges. Research has shown that such interventions are more effective when they are culturally-sensitive and delivered to communities, rather than to individuals or families (66). In the late 1990's, Canvasback Missions, Inc., recognized the need for such interventions in the RMI and conducted two lifestyle intervention programs with excellent results, using a modified version of the NEWSTART® wellness program operated by the Guam Seventh-day Adventist Clinic (67). The program emphasizes a whole-food, plant-rich diet with moderate exercise and is used to treat T2D, obesity, and other chronic diseases. Although no studies have been published on the intervention, Guam physicians have found it effective and continue to refer their patients to wellness centers (68).

Building on the success of these lifestyle intervention programs in Guam and the RMI, we designed and conducted the first randomized controlled trial to rigorously evaluate whether a comprehensive, culturally-sensitive, lifestyle intervention program can treat T2D in the RMI. The goal of the study was to determine the effectiveness of an intensive lifestyle intervention consisting of a mostly plant-based diet and regular moderate exercise in Marshall Islanders with T2D. The present study was also the first randomized clinical trial ever conducted in the RMI and the first lifestyle intervention trial ever conducted in Micronesia. The primary aim was to determine whether the lifestyle intervention can improve glycemic control. The primary endpoints included fasting glucose, fasting insulin, HbA1c, insulin resistance as measured by the Homeostatic Model Assessment of Insulin Resistance (HOMA-IR), and usage of diabetes medications. The secondary aim was to determine whether the lifestyle intervention could improve cardiovascular risk factors, including lipids, blood pressure, heart rate, high-sensitivity C-reactive protein (hs-CRP), body weight, body mass index (BMI), and waist circumference. We hypothesized that study participants would be able to make sufficient lifestyle changes—including by modifying their diet and increasing their physical activity—to achieve clinically meaningful improvements in glycemic control and cardiovascular risk factors, relative to the control group.

## Materials and Methods

### Study Design

#### Study Overview

This trial was conducted in partnership with the RMI's Ministry of Health (MOH). The study was designed as an open-label, parallel-arm, randomized controlled trial and was conducted at the MOH's Diabetes Wellness Center (DWC) in the city of Majuro in the RMI. Adults with T2D were randomized to receive either the standard of diabetes care (control group) or a lifestyle intervention comprising a whole-foods, largely plant-based diet combined with increased physical activity (intervention group). The intervention group received intensive counseling, support, and group sessions over a 12-week period and then continued the lifestyle intervention on their own for the remainder of the 24-week intervention. Health outcomes were assessed at weeks 0 (baseline), 2, 6, 12, and 24. At the end of the study intervention, those who were randomized to the control group were offered the option to crossover to the experimental arm and complete the lifestyle program. The study was approved by the Loma Linda University Institutional Review Board (IRB #: 59105) and an *ad-hoc* Institutional Review Board in the RMI that was set-up specifically for the trial. The study was conducted in accordance with the Declaration of Helsinki. All participants provided written informed consent prior to enrolling in the study and were given a stipend for their participation. Due to the nature of the intervention, the study was not blinded.

#### Recruitment

During year 1 of the study, participants were initially recruited from the general public. Two local leaders assisted in building interest among and recruiting Majuro residents. During year 2, participants were recruited both from the general public and from the MOH Diabetes Clinic through its diabetes registry. Of those screened, 18% or 31 out of 169 eligible participants dropped from the trial before beginning the intervention. The first cohort began the study intervention in June 2006 and the last cohort began in July 2008. Due to a lack of funding after the clinical trial ended, the study data was not analyzed and published until the present.

#### Eligibility Criteria

Recruited individuals were screened against a series of inclusion and exclusion criteria. The inclusion criteria were: (1) resident of RMI, (2) aged 18–75 years, (3) HbA1c ≥ 8.0% or diagnosed with T2D and taking diabetes medication, and (4) medical clearance to participate from DWC physicians. The exclusion criteria included (1) recent ( ≤ 3 months) change in a diabetes-related medication dosage, (2) a physical or medical condition that would impede participation in the lifestyle intervention (e.g., wheel-chair bound, unstable angina), (3) evidence of significant coronary heart disease, and (4) previous participation in an intensive lifestyle intervention. There were no eligibility criteria related to BMI.

#### Screening

Prospective participants were screened during a single in-person visit, which included a review of their medical history, physical fitness testing, a dietary assessment based primarily on a food frequency questionnaire, and fasting blood draws to measure glucose and HbA1c. Screening was performed at the DWC. To ensure suitability for the exercise portion of the intervention, participants were queried about their cardiovascular fitness and history. Those with ischemic heart disease were required to have special clearance from their physician. A physician reviewed all dietary, medication, and medical history data for medical clearance. Individuals who passed the screening process later attended an oral presentation explaining the study. During these group sessions, the study was explained to the participants in detail. Translators were assigned by the MOH to explain the study to participants who did not understand English. Interested participants then read and signed the informed consent in English or their native language.

#### Randomization

Following enrollment, participants were randomized to the two groups. Spouses who enrolled in the trial were randomized in pairs to keep them together. Non-eligible spouses were encouraged to attend counseling sessions but were not enrolled in the study.

#### Cohorts

Participants were enrolled in five overlapping cohorts, with each cohort including both a control and intervention group (described below). Within each cohort, participants were randomized to one of the two groups in approximately equal numbers. The cohorts were spread over a two-year period, with Cohorts 1 and 2 finishing in year 1 and Cohorts 3–5 finishing in year 2. The cohorts 1–5 consisted of 27 (13 in the intervention group vs. 14 controls), 41 (21 vs. 20), 31 (9 vs. 22), 27 (14 vs. 13), and 43 (22 vs. 21) participants, respectively. The control arm received the usual standard of diabetes care, while the intervention arm received a lifestyle intervention. Unfortunately, not all participants in the control group in year 1 sought and received the usual standard of care for diabetes treatment as instructed, so starting in year 2, participants were primarily recruited from the MOH Diabetes Clinic, in order to ensure that the control group was compliant with the standard of care. Furthermore, to improve the delivery of the intervention, Cohorts 3–5 received an enhanced version of the lifestyle intervention. The enhanced version was identical to the standard version of the intervention, with the exception of more patient education and contact during weeks 4 and 6 of the study. The nature of the dietary and exercise intervention itself was not changed and did not vary across cohorts.

### Study Intervention

#### Usual Diabetes Care

The control group received the standard of diabetes care in the RMI, which consisted of the standard treatment protocols used by the MOH Diabetes Clinic. These included placing T2D patients on anti-hyperglycemic agents appropriate to their HbA1c levels (including sulfonylureas, metformin, and insulin) and providing oral and written information about the importance of maintaining a healthy weight, eating a healthy diet, and getting regular exercise. Participants in the control group were instructed not to make changes in their diet and activity levels during the study.

#### Lifestyle Intervention

The lifestyle intervention consisted of a high-fiber, low-fat, mostly plant-based diet, and moderate exercise. Participants initially received 12 weeks of group educational classes and meals prepared by the DWC Wellness Kitchen and then followed the lifestyle intervention on their own for the remaining 12 weeks (Table 1). Group classes included informative sessions on healthy eating, exercise (both aerobic and strength training), and stress management, as well as hands-on cooking classes. Classes were delivered as a combination of PowerPoint presentations, practical workshops, dine-outs, shopping tours, and cooking classes with spouses. To foster access to affordable produce, participants were also taught by soil and gardening experts how to grow their own vegetables and were taken on agricultural field trips.

**Table 1:** Standard and enhanced versions of the intensive lifestyle intervention.

**Standard version**	**Enhanced version**	**Frequency**	**Meals/day**	**Group classes**	**Exercise**
Weeks 1–2	Weeks 1–2, 4, and 6	4 days/week	3	5–6 h	1 h
Weeks 3–6	Weeks 3 and 5	2 days/week	1	4–5 h	30–60 min
Weeks 7–12	Weeks 7–12	1 day/week	1	4–5 h	30–60 min
Weeks 13–24	Weeks 13–24	1 day/week	0	Turn in Pedometer Readings	

*The standard and enhanced versions of the lifestyle intervention differ only during weeks 4 and 6*.

**Figure 1:**
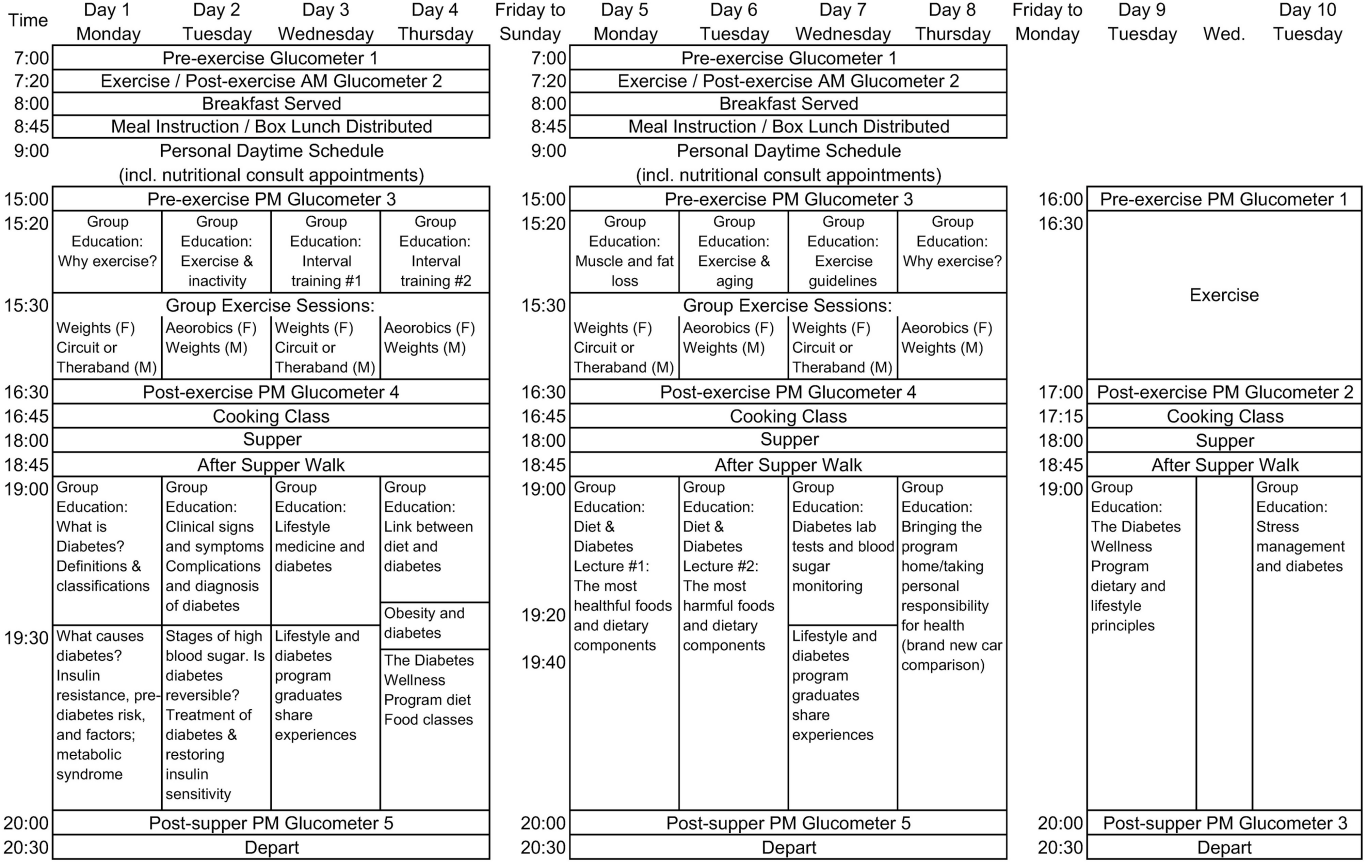
Schedules for the first 3 weeks of the lifestyle intervention.

The initial 12-week program was structured as follows. During the first 2-week intensive phase, participants in all cohorts attended 5–6 h/day of group classes on Mondays–Thursdays (4 days/week) at the DWC, totaling 36 h of health education over the first two weeks. Figure 1 displays the schedule for the intensive phase. On clinic days, group sessions included 1 h/day of exercise, and participants were fed 3 meals/day made by the DWC Kitchen, according to the menus shown in Table 2. Participants ate breakfast at the DWC and took lunch boxes with them to eat at work or at home. The participants then returned to the DWC for a 5-h session from 3:00 to 8:00 p.m., where they helped prepare the meals and then ate dinner. Participants prepared the main dish, and the kitchen staff made the side dishes (e.g., green salad). During weeks 3–6, participants who received the standard version of the intervention (Cohorts 1–2) spent 4–5 h twice per week (Tuesdays and Thursdays) in group sessions, including 60 min/day of structured exercise, a 10–20-min walk after each meal, and also consumed an evening meal provided by the DWC Wellness Kitchen (see menus in Table 3). For those receiving the enhanced version of the intervention (Cohorts 3–5), weeks 4 and 6 of the standard intervention were replaced with weeks 1 and 2 to increase the intensity of instruction, as shown in Table 1. During weeks 7–12 of the program, participants in all cohorts participated in a 4–5 h once per week session with structured exercise and group classes and were provided an evening meal. Finally, during the last 12 weeks of the trial (weeks 13–24), no instruction or counseling was provided, and participants came to the center weekly to turn in their pedometer readings.

**Table 2:** Study menus for weeks 1–2.

	**Breakfast**	**Lunch**	**Dinner**
**WEEK 1**			
Monday	Not provided	Salad bar Sesame tahini dressing Pumpkin and/or breadfruit and red bean salad Cream of broccoli soup	^*^Crispy tofu fingers ^*^Spicy eggplant Fresh fruit or vegetables
Tuesday	^*^Pineapple and coconut muesli ^*^Beans and greens Ground flax Soymilk Jelée parfait	Salad bar Italian dressing Five-bean salad Spiral noodle salad Pea soup	^*^Garbanzo à la king Fresh fruit or vegetables
Wednesday	^*^Kamut/barley cereal Beans and greens Walnuts Ground flax Soymilk ^*^Jelée parfait	Gado gado Savory tofu Steamed breadfruit cubes Bean and barley soup	^*^Papaya or pumpkin stew Fresh fruit or vegetables
Thursday	^*^Oatmeal ^*^Crunchy granola Ground flax Beans and greens Soymilk Jelée parfait	Salad bar Creamy dill dressing Sesame crackers “Eggless” egg salad Hummus Lentil soup	^*^Chili Fresh fruit or vegetables
Friday	^*^Creamy barley cereal ^*^Easy beans/greens Walnuts Ground flax Soymilk Jelée parfait	Not provided	Not provided
**WEEK 2**			
Monday	Not provided	Salad bar Yam, black bean, and greens salad with balsamic vinaigrette Cabbage soup	^*^Garbanzo kelel ^*^Cabbage salad Fresh fruit
Tuesday	^*^Apple raisin muesli Ground flax Easy beans/greens Soymilk Jelée parfait	Salad bar Thousand Island dressing Rice and lentil salad Black bean and corn soup	^*^Ma stew Fresh fruit or vegetables
Wednesday	^*^Creamy barley cereal Walnuts Ground flax Beans and greens Soymilk ^*^Jelée parfait	Salad bar Sesame dressing Thai noodle salad Oriental cabbage salad Eggplant black-eyed pea soup	^*^Tofu or vegetable stir fry ^*^Long beans in black bean sauce Fresh fruit or vegetables
Thursday	^*^Multigrain cereal Ground flax Beans and greens Soymilk Jelée parfait	Tropical haystacks (rice, pinto beans, lettuce/cabbage, peppers, peanuts, coconut, and pineapple) Golden sauce Pumpkin ginger soup	^*^Lentil loaf ^*^Mushroom gravy ^+^Roasted vegetables Fresh fruit or vegetables
Friday	^*^Scrambled tofu with peppers, onions, and greens Easy oat burgers Jelée parfait	Not provided	Not provided

* *Recipes prepared by participants*.

+ Recipes demonstrated to participants

**Table 3:** Study menus for weeks 3–12.

	**Tuesday dinner**	**Thursday dinner**
Week 3	Green salad ^+^Balsamic vinaigrette ^*^Creole ^+^Barley	Promotion night Finger food provided by Diabetes Wellness Center, including breadfruit wedges, and taro squares
Week 4*(standard version only)*	Green salad ^*^Thousand Island dressing ^*^Vegetable and bean stew	Green salad ^+^Italian dressing ^*^Pasta primavera ^+^Spicy roasted chickpeas
Week 5	Potluck and/or games night (all groups)	^*^Red lentil soup ^*^Mexi dip ^+^Pita wedges Raw vegetables ^+^Banana bread
Week 6*(standardversion only)*	Cucumber salad Wet pumpkin curry Brown rice ^+^Banana ice cream	Green salad Balsamic vinaigrette ^*^Bean burger ^+^Whole-wheat bread or rolls
**ONCE-A-WEEK DINNER**		
Week 7	Green salad ^+^Tahini dressing ^*^African stew ^+^Barley	
Week 8	Green salad Italian dressing ^*^Polynesian beans and vegetables ^*^Polynesian fruit bars	
Week 9	Asian salad Sesame or ginger dressing ^*^Tofu triangles with mushrooms ^*^Brown rice	
Week 10	Papaya salad ^+^Coconut mayonnaise ^*^Stewed beans ^*^International greens ^*^Cornbread	
Week 11	Green salad ^+^Creamy dill dressing ^*^Tamale pie ^+^Brownie bars or banana carob cake	
Week 12	Green salad ^+^Oil and vinegar dressing ^*^Sweet and sour tofu Brown rice ^+^Peru pudding	

*The menus for weeks 3–12 were the same for both the standard and enhanced versions of the intensive lifestyle intervention, with the exception that the weeks 1 and 2 menus were used for weeks 4 and 6, respectively, of the enhanced version of the intervention*.

* *Recipes prepared by participants*.

+ *Recipes demonstrated to participants*.

#### Diet

The dietary intervention was adapted from the NEWSTART® program (67), a plant-based diet used by the Guam Seventh-day Adventist Clinic Wellness Center, and from concepts presented in the book *Defeating Diabetes* (69). (NEWSTART® stands for Nutrition, Exercise, Water, Sunshine, Temperance, Air, Rest, and Trust in divine power.) The diet used in this trial was mostly plant-based and emphasized whole plant foods, high fiber intake (roughly 35 g fiber per 1,000 kcal), low fat intake (20–25% of energy intake), moderate protein intake (10–15% of energy intake), and moderate sodium intake (< 2,400 mg/day) while encouraging local cuisine (70). The diet was designed to be high in phytochemicals and antioxidants, low in dietary oxidants, and to have a low glycemic load. Concentrated fats and oils were minimized, and only fat from healthy sources was permitted. Saturated fat was limited to < 7% of caloric intake, and no trans fatty acids were permitted. The diet included sufficient omega-3 fatty acids sourced from plants (primarily ground flaxseeds and walnuts), and fish, for those who chose to include it at home after the 2-week intensive phase of the intervention.

In the first 2 weeks of the dietary intervention (the intensive phase), participants consumed a 100% plant-based diet that minimized ground grains (e.g., flour, pasta, bread) and refined carbohydrates (e.g., refined sugar and starches). Also during the intensive phase, participants consumed 12 meals/week that were prepared for them by the DWC's Wellness Kitchen (see menus in Table 2), and they were instructed not to eat any outside food or snacks the remainder of those days. After the 2-week 100% plant-based phase, participants were allowed to consume boiled, steamed, or grilled fish or seafood if desired, and they continued to eat a smaller number of meals provided by the DWC (see Tables 2, 3).

When not eating meals prepared by the DWC, participants were counseled to make healthy food choices on their own using a tiered food classification system, with foods classified into four groups: 1st, 2nd, 3rd, and 4th classes (Table 4). Participants were encouraged to eat freely from the 1st class, moderately from the 2nd class, and sparingly from the 3rd class, and to completely avoid items in the 4th class. The food classification system encouraged participants to eat mostly first-class foods (75–100% of daily calories), which were defined as unprocessed, whole plant foods. To maximize nutrient density and reduce glycemic load, the first class category emphasized non-starchy vegetables, legumes, and foods rich in viscous fiber (e.g., ground flaxseed, psyllium, oats, barley, beans, and some vegetables and fruits, such as sweet potatoes, local pumpkin, turnips, and citrus fruits). Second-class foods—defined as minimally processed products such as non-dairy milks, meat substitutes, canned vegetables, and whole grain flour products—were permitted but used less often. Third-class foods included moderately processed foods, such as processed whole grains (e.g., flaked and puffed cereals) and white flour products, and animal products with moderate fat content. Fish that was not fried was permitted after the intensive phase of the program, and non-fish animal products—such as poultry, eggs from local chickens, and lower-fat dairy—were permitted, but participants were encouraged to minimize consumption of these foods. Fourth-class foods—such as beverages with added sugar, processed meat, fried foods, fast foods, and confections—were prohibited. Within all food categories, lower glycemic index foods were selected. For example, barley was emphasized in the grain group, as it has the lowest glycemic index of all grains. Portions of concentrated carbohydrate foods such as whole grains and starchy vegetables were controlled, and portion sizes of very high-fat foods such as nuts, seeds, and coconut were small to help keep calories controlled.

**Table 4:** Tiered food classification system.

	**1ST CLASS FOODS: Whole Plant Foods (75–100% of calories)**	**2ND CLASS FOODS: Lightly Processed Foods and Low-Fat Animal Products (≤25% of calories)**	**3RD CLASS FOODS: Moderately Processed Foods and Mid-Fat Animal Products (≤10% of calories)**	**4TH CLASS FOODS: Heavily Processed Foods and High-Fat Animal Products (≤5% of calories)**
Vegetables	Green leafy vegetables Non-starchy vegetables: broccoli, carrots, cauliflower, onions, cucumbers, egg plant, peppers, mushrooms, tomatoes, sprouts Starchy vegetables: potatoes, sweet potatoes, corn, winter squash	Green leafy vegetables that are canned or cooked in oil	Battered vegetables Vegetables in high-fat sauces	Vegetable chips French fries Deep-fried potatoes or sweet potatoes
Protein foods	Legumes: beans, lentils, peas	Tofu Tempeh Fish (not fried)	Lean meat Poultry (no skin) Eggs Fish canned in oil	Processed or canned meat (e.g., bacon, sausage, cold cuts, frankfurters) High-fat meat Poultry Fried meat
Grains and cereal	Whole grains: barley, rye, kamut, spelt, brown rice, quinoa Cut and rolled grains	Slightly processed whole-grain breads and cereals (incl. whole wheat or other whole grain flour)	Moderately processed grains (e.g., granola bars, white flour, pasta, bagels, white rice)	Heavily processed, white flour products (e.g., donuts, cookies, and other sweet baked goods) Corn chips
Fruit	Fresh, dried, and frozen fruit	Lightly processed fruits (e.g., canned fruit in natural juice)	Fruit canned in syrupFruit juice	Fruit jams and jellies Fruit-flavored punch and drinks
Dairy/dairy alternatives	Fresh pressed nut and seed milks	Soymilk (unsweetened or original) Other commercial non-dairy milk (almond, hemp, rice)	Non- or low-fat dairy products (incl. skim or low-fat milk and cheese)	High-fat dairy products (e.g., whole milk, cream, cheese, ice cream, sour cream, whipping cream)
Higher fat whole foods, fats, and oils	Nuts, seeds and their butters (natural) Avocados Olives	Nuts and seeds with small amounts of added oil or salt Nut butters with sugar or salt	Vegetable oils Zero trans-fat margarines Nut butters with hydrogenated fats Nuts with fat, sugar, and salt	Lard Shortening Butter Hydrogenated margarine
Other			Natural sweeteners (e.g., maple syrup, blackstrap molasses, honey, brown rice syrup)	Refined sugar (e.g., white sugar, syrup) Soda Sugar-sweetened beverages Candy

#### Exercise

Participants were asked to exercise at least 30–60 min per day throughout the study, which included both solo exercise and structured group exercise classes that decreased in intensity as the study proceeded (Table 1). The structured group exercise included culturally acceptable physical activities as determined by focus groups. Group exercise included walking, calisthenics, elastic-band resistance training, Marshallese-style dance classes, and use of gym equipment, such as treadmills, cardio gliders, rowing machines, and free weights. During the 2-week intensive phase of the intervention, participants attended a 1-h group exercise class 4 days per week and completed 2 daily walks on their own, with one before breakfast and one after lunch. They also completed one daily walk as a group after dinner. Each after-meal walk was 10–20 min. On non-clinic days, participants exercised solo. They were encouraged to exercise at the clinic when possible, and when not possible, they were asked to use their therabands and to exercise for at least 30–60 min at home. After the 2-week intensive phase, participants attended 60-min exercise group classes on clinic days and were counseled to exercise on their own on non-clinic days. They were also advised to continue with their daily walks (including 10–20 min walks after each meal) and to attend group fitness classes (such as dance classes).

#### Stress Management

Participants in the intervention group were also provided with stress counseling and management activities, such as deep breathing, stretching, and words of encouragement. They were provided a lecture on suggestions for handling stressful situations and getting support.

#### Cultural Adaptation

The intervention was delivered in a culturally-sensitive manner by trained indigenous Marshallese and Canvasback Missions staff. Prior to designing the intervention, Canvasback Missions had more than 20 years of experience working with the Marshallese people and government, including operating NEWSTART lifestyle programs. The intervention was designed in partnership with the RMI MOH. The study team met with the MOH and health care providers to determine the appropriate level of content and preferred methods of learning. Consequently, the program was structured to be interactive and rely on active learning. All Canvasback Missions staff were provided with significant cultural sensitivity training that included instruction in the cultural traditions and local customs of respect. Marshallese staff were hired, trained, and performed as many study functions as possible, including meal preparation and group activities. Training included a trip to Guam to learn the NEWSTART program before coming back to Majuro to set-up the study. We also engaged local physicians, nurses, dance instructors, and our staff cooks to be a part of education sessions, in an effort to make the program culturally relevant. This was supported by strong relationships that were forged with Marshallese and American dignitaries, the MOH personnel, community group leaders, and store managers. Study documents, lectures, and instructions were provided in the Marshallese language. To tailor the dietary intervention, recipes from Marshallese cuisine were incorporated and adapted while still ensuring that the diet was plant-based and met nutritional targets. Finally, group exercise activities were led by a Marshallese staff member who was known in the community for leading such activities, and local dance routines were encouraged as aerobic activity.

### Measurements and Outcomes

#### Assessments

Study assessments included demographics, anthropometrics, vital signs, serum cardiometabolic risk factors, medication usage, and behavioral assessments. All study assessments were performed at weeks 0, 2, 6, 12, and 24 unless otherwise stated below.

#### Anthropometrics and Vital Signs

Height, weight, waist circumference, blood pressure, and resting heart rate were all measured in duplicate using standard procedures. Height was measured using a stadiometer and was used to tabulate BMI. Waist circumference was measured at the level of the umbilicus above the iliac crest.

#### Serum Chemistry

Serum analytes included HbA1c and fasting glucose, insulin, lipids, and hs-CRP. Lipids included total cholesterol, HDL cholesterol, and triglycerides, with LDL cholesterol calculated using the Friedewald equation. Analytes were measured using the CLIA-approved laboratory at the MOH's Hospital. HOMA-IR, which is a product of fasting glucose and fasting insulin, was used to estimate insulin resistance. LDL cholesterol values above 400 mg/dl will be considered unreliable and be replaced with the maximum reliable value of 400 mg/dl. hs-CRP values above 10 mmol/l were taken as indicative of an active acute infection, rather than as measures of chronic low-grade inflammation, and were therefore treated as missing values.

#### Medications

To monitor changes in glucose levels in the intervention group and adjust medication dosages accordingly, a glucometer was used during the first 2 weeks and fasting labs at weeks 2, 6, and 12 were relied on thereafter. Medication dosages were adjusted by participants' primary care physicians and/or the DWC clinicians. Medications and their dosages were recorded at each assessment. Changes in the average number of medications and in the percentage of participants taking one or more medications will be compared across groups for both all medications and diabetes-specific medications.

#### Glucometers

Participants in the intervention group were given glucometers during the intensive 2-week phase of the intervention to measure their glucose levels, as needed. These assessments were used both for educational purposes and to make medication adjustments.

#### Dietary Assessment

Participants completed a food frequency questionnaire at baseline (week 0) and at week 24. The questionnaire was tailored to Majuro residents and asked participants about the frequency with which they consumed 110 food items, as well as the typical serving size and any seasonal differences in consumption (for fresh fruits and vegetables only). Participants also described what they usually ate for breakfast, lunch, dinner, and snacks. Participants also completed a one-page, self-reported diet quality assessment at weeks 2, 6, and 12 to report any changes in food intake, including the foods they now avoid, eat less of, and eat more of. Changes in dietary intake and other lifestyle factors were used to help gauge the program's effectiveness. At the end of the trial, participants also self-reported their adherence to the diet using a 1–5 Likert scale.

#### Physical Activity Assessment

Cardiopulmonary fitness was assessed at each time point using the Harvard step test in year 1 and both the Harvard step test and the timed 1-mile walk in year 2. For the Harvard step test, participants stepped up onto a gym bench ~20 inches in height once every 2 s for 5 min or until the participant could not maintain the pace. Thereafter, they were seated in a chair, and their pulse was assessed 1, 2, and 3 min after completing the step tests, and fitness level was graded as 50 × (duration in seconds)/(sum of the three post-exercise heart rates). For the timed 1-mile walk test, potential participants were given pedometers to wear and were asked to walk a one-mile roadway loop outside the hospital at a pace they could maintain without running or stopping. Participants were instructed to walk alone at their own pace. After the individuals returned, their walking time and steps were collected and recorded. Physical activity levels were assessed by self-report using a modified version of the International Physical Activity Questionnaire at each time point and through weekly pedometer readings**. **All participants—including those in the control group—were asked to wear pedometers throughout the study and report their step count on a weekly basis. Participants came to the DWC to report their readings or, if they were unable to come in, called study staff to report their readings.

#### Safety

Participants were instructed to report adverse events as soon as they occurred and were also queried about adverse events at each assessment. To minimize the risk of complications from exercises, warm-up exercises were used to gently increase the intensity of the physical activity.

## Data analyses

### Statistical Power Calculation

Since the trial proceeded in phases, the initial data collected in year 1 was used to determine the overall sample size needed for the trial. Based on the variances observed in the primary endpoints in the year 1 cohorts, a sample size of *N* = 120 in total was needed to provide at least 80% power (two-sided, α = 0.05) to detect between-group differences of 20 mg/dl in fasting glucose and 1.0% in HbA1c.

### Statistical Analyses

All data will be analyzed using two-sided statistical tests and a Type I error rate of α = 0.05. The intention-to-treat method will be used for the primary analyses but will be compared to outcomes from actual-treatment-received analyses (i.e., per protocol analyses). Categorical data will be compared between the two groups using chi-square or Fisher's exact tests. Continuous data will be compared using linear mixed models or non-parametric tests (as appropriate), with potential adjustments for changes in medication dosages and potential covariates such as baseline values, age, biological sex, and cohort number. Likelihood functions will be examined to determine the appropriateness of covariates and to avoid overfitting the data. Multivariable linear and logistic regression will be used to identify predictors for improved health outcomes. Lastly, subgroup analyses may be performed by sex, age, co-morbidity, length of diagnosis, and severity to evaluate heterogeneity in treatment responses.

## Discussion

This study leverages interventions that are clinically effective in an already-established wellness program in Guam and tested them for the first time in a randomized controlled trial. The main goal of the study was to determine the effectiveness of an intensive lifestyle intervention consisting of a mostly plant-based diet and regular moderate exercise in Marshall Islanders with T2D. We expected that study participants would be able to adopt a plant-based diet and engage in moderate exercise sufficiently to achieve clinically meaningful improvements in glycemic control and that many participants would reduce their reliance on diabetes medications and/or even potentially go into remission from T2D. We also expected that participants would experience improved lipid profiles, reduced blood pressure, and lower levels of inflammation, relative to the standard of care. To support these objectives, we leveraged ongoing organizational networks within the RMI, including Canvasback Missions' more than 11 years of experience in providing medical services to T2D patients in the RMI. Moreover, we forged partnerships with the local government to deliver a culturally-adapted version of an intensive lifestyle intervention using trained indigenous staff.

Through implementing this clinical trial, we achieved several major community-wide successes, which serve as a model for future lifestyle intervention trials and community-based participatory research. One of the major achievements is that we assisted in designing an expanded and improved 6,700-square foot Diabetes Wellness Center, which has now been established and is operating in Majuro. The intervention itself was so successful that supermarket buying patterns in Majuro have changed. Reports from the local supermarket indicate that the demand for healthy foods has increased to such an extent that these items are “gone in 2–3 days.” The DWC received many requests to speak and present intervention programs for churches, schools, and service groups. The DWC also held a seminar for the members of the Nitijela (Parliament) and the president's cabinet. As a result of the influence of these leaders, the community has begun to respond by changing their food choices and exercising more. When DWC staff first arrived in Majuro, walking was frowned upon, and people tended to take taxis, even for short distance outings. Now, walking for exercise has become sufficiently popular that there are concerns for public safety, as most walking takes place on the roads. As a result, the mayor and police commissioner have agreed to close down the main highway in the early morning to provide the community with a safe place to walk during the cooler dawn hours. More markets are setting aside sections for health foods, and the island now has a good selection of these foods. Produce is being flown in to meet the new public demand. DWC-approved items are appearing on local restaurant menus. The MOH has asked Canvasback Missions to take over food preparation for the hospital. The Department of Education approached Canvasback Missions to develop a nutrition curriculum for grades K-6 in public schools in Majuro and to train teachers. Canvasback Missions was awarded a grant from the World Diabetes Federation. Brenda Davis, RD and Margie Colclough, SLP, were recently brought into Majuro to complete this work. They developed a comprehensive 13-module curriculum called *Get Healthy at School*. Teachers in all nine public schools in Majuro were trained to use these materials in their classrooms. The MOH also held a 2-day conference with its senior administrative staff and leading doctors to find ways to focus on preventive health care. Word of the success of this program has reached many of the surrounding Pacific Island jurisdictions, and the DWC often hosts visitors from these countries.

In achieving these successes, we overcame several challenges. First, recruitment was slower than expected in year 1, so in year 2, we increased enrollment rates by recruiting subjects from the MOH Diabetes Clinic. Another benefit of this change in our recruitment strategy is that it improved compliance to the standard of care in the control group. During year 1, when participants were screened from the general public, they did not always adhere to the standard of care. Recruiting subjects who were already receiving the usual care and regularly met with their physicians resolved this problem. In year 2, as the word spread throughout the country that the DWC program was controlling and reversing T2D naturally, more and more people came forward and wanted to participate, and recruitment was no longer a challenge. In fact, by the end of the trial, more than 100 people were on the waiting list for the DWC program. Another challenge faced in year 1 is that about 25% of the adults screened did not comprehend English well enough to understand materials presented in English. This may have introduced selection bias by enrolling better-educated participants in year 1. Therefore, in year 2, all program materials were translated into Marshallese and presented in Marshallese, initially by translating English presentations, and then later by replacing English presentations with Marshallese presentations. Cohort 5 was the first all Marshallese-run program. After an initial hurdle of training local staff, who had little previous experience and limited education, to become precise and accurate recordkeepers and accurate presenters, we were able to deliver an all Marshallese-version of the intervention.

As for the intervention itself, we observed that the 4-times-per-week intensive phase appeared to be highly effective in promoting adherence to the lifestyle intervention and improving glycemic control. However, once the intervention intensity dwindled to once-every-other-week, a majority of participants did not maintain adherence as well, so adherence waned in the later stages of the trial. This may partially be due to the fact that intensive lifestyle changes can be socially and culturally isolating, particularly because the RMI is a highly social and culturally engaged society, where food is often a central part of cultural events. After some trial and error, we noticed that moving from a 4-times-per-week intensive phase to a twice-per-week phase (rather than every other week) is a more effective approach. It is important for future trials on intensive lifestyle interventions to continue testing the optimal intensity of lifestyle interventions. One of the main unanticipated effects of the intervention is that a significant fraction of the control group attempted the intensive lifestyle program on their own, despite being randomized to the standard of care. Such participants were spotted purchasing foods, such as oatmeal and flaxseeds, at the grocery store, despite not having used these foods before; some of these participants admitted that they were trying to do the lifestyle intervention on their own. In total, we estimate that about one-fourth of the controls adopted significant lifestyle changes during the 24-week intervention, despite instructions to the contrary and despite having the opportunity to crossover to the intervention group after completing the control arm. Contamination of the control group tends to bias outcomes toward the null hypothesis, so our anticipated future results may underestimate the actual treatment effects. Another intervention-related concern is the potential for nutritional shortfalls, especially regarding vitamin B12. Although we used some vitamin B12-fortified foods, such as nutritional yeast and fortified soymilk, such fortified foods are not as readily available in the RMI, and there was concern that over time, vitamin B12 intakes could be inadequate, especially for participants taking metformin. Also, healthy foods, such as fruits and vegetables, are expensive in the RMI, and residents tend to have few resources.

The management of adverse events and data collection in a country with no prior research infrastructure presented the greatest challenges to the trial. The main challenge for adverse event management was adjusting medication dosages downwards to avoid hypoglycemic reactions in the intervention group. When participants were not in the clinic, they often had to return to their family physician to adjust their medication dosages, which meant long wait times and a fee. This was particularly problematic for participants on insulin, who had to be very carefully monitored so that insulin dosages could be reduced or stopped accordingly. Further, because medication dosing tended to increase for control subjects and decrease for intervention subjects, future reported outcomes will likely be biased toward the null hypothesis. We also faced problems with missing data due to participants not showing up for appointments, procedural failures by the laboratory, and the hospital being new to the extensive documentation requirements for clinical trials, in a country where clinical trials have never been performed before. It took several trips and digging through boxes of duplicate lab slips to fill in missing logbook entries. The positive outcome is that new policies and reporting procedures were established for any future lab work that may be required, and all lab work that was done is now backed up. To tackle the challenge of participant attendance at clinic visits, research staff attempted to reach every participant by phone, and in some cases, went to the participant's home or work to remind them of the importance of the blood draws and measures to complete the study. We have discovered that within the RMI, more effort is needed than in the U.S. to ensure that participants fully participate in the intervention.

Overall, though, the clinical trial was a success. We delivered a novel lifestyle intervention, which constituted the first randomized clinical trial conducted in the Republic of the Marshall Islands; the first lifestyle intervention trial conducted in Micronesia; and the second clinical trial to investigate the effects of a plant-rich diet combined with moderate exercise in T2D patients. We intend to publish future manuscripts reporting the results of the clinical trial. We expect that the results of this study will help guide future medical care in the Pacific Islands and provide insight into how to best direct resources to treat indigenous populations with T2D. Outside of the Pacific Islands, it will shed light on how to effectively deliver comprehensive, intensive lifestyle interventions for the treatment and management of diabetes. Finally, it will provide critical insight into the degree to which lifestyle interventions can treat and reverse type 2 diabetes.

## Ethics Statement

The study was approved by the Loma Linda University Institutional Review Board and an *ad-hoc* Institutional Review Board in the RMI that was set-up specifically for the trial. The raw data may be requested by contacting the corresponding author, Dr. John Kelly, at jhkelly@bhhec.org, after the study results are published. The data request will be reviewed by the RMI Ministry of Health.

## Author Contributions

JK designed the study, with input from JS. BD, RH, and JK conducted the study. HJ, CP, and RK performed the analyses and drafted the manuscript. All authors revised and approved the final version of the manuscript.

### Conflict of Interest Statement

BD is the author of the books Defeating Diabetes and The Kick Diabetes Cookbook: An Action Plan and Recipes for Defeating Diabetes. The remaining authors declare that the research was conducted in the absence of any commercial or financial relationships that could be construed as a potential conflict of interest

## Abbreviations

T2D: Type 2 diabetes
RMI: Republic of the Marshall Islands
BMI: body mass index
HbA1c: hemoglobin A1c
HOMA-IR: Homeostatic Model Assessment of Insulin Resistance
hs-CRP: high-sensitivity-C-reactive protein
MOH: Ministry of Health
DWC: Diabetes Wellness Clinic
NEWSTART: Nutrition, Exercise, Water, Sunshine, Temperance, Air, Rest, and Trust in divine power.
